# Therapeutic effects of HESA-A (a herbal-marine compound) in acute organophosphorus pesticide poisoning

**Published:** 2020

**Authors:** Seyed Reza Mousavi, Mohammad Moshiri, Emadodin Darchini-Maragheh, Seyed Khosro Ghasempouri, Bita Dadpour, Faezeh Sardar Antighechi, Mahdi Balali-Mood

**Affiliations:** 1 *Medical Toxicology Research Center, School of Medicine, Mashhad University of Medical Sciences, Mashhad, Iran. *; 2 *Cutaneous Leishmaniasis Research Center, Emam Reza Hospital, School of Medicine, Mashhad University of Medical Sciences, Mashhad, Iran.*; 3 *Department of Forensic Medicine and Toxicology, School of Medicine, Mazandaran University of Medical Sciences, Sari, Iran.*; 4 *Imam Reza Hospital, Mashhad University of Medical Sciences, Mashhad, Iran.*; 5 *Medical Toxicology and Drug Abuse Research Center, Birjand University of Medical Sciences, Birjand, Iran.*

**Keywords:** Organophosphorus pesticides Poisoning, Clinical trial, HESA-A

## Abstract

**Objective:**

Organophosphorus compounds (OPs) are common causes of poisonings. Atropine and oximes are pharmacological antidotes of OPs. However, because of their adverse effects and insufficient performance, several other compounds have been evaluated as adjuvant therapy. HESA-A is a herbal-marine drug that contains material from *Carum carvi* (Persian cumin), *Penaeus latisculatus *(king prawn), and *Apium graveolens* (celery) with anti-inflammatory and antioxidants properties, which has shown useful effects as adjuvant therapy on some diseases. We have evaluated the effect of HESA-A on 69 moderate to severe acute OPs poisoned patients (44 HESA-A treated and 25 controls) as an adjuvant drug.

**Materials and Methods:**

Two randomized age and sex matched groups of OPs poisoned patients were treated in Medical Toxicology Center of Imam Reza hospital, Mashhad, by conventional therapy with or without HESA-A (50 mg/kg/day orally). The evaluation criteria were total administrated doses of atropine and pralidoxime, intensive care unit (ICU) admission rate, mechanical respiration need, number of hospitalization days and mortality.

**Results:**

There were no significant differences between the morbidity and mortality rate criteria of the two groups; moreover, we did not observe significant adverse effects for HESA-A.

**Conclusion:**

HESA-A did not reduce morbidity and mortality of OPs poisoning and did not induce any major side effect in the patients.

## Introduction

Organophosphorus compounds (OPs) are used as pesticides and chemical warfare agents (Moshiri et al., 2012 ▶). Good performance and environmental instability make them the most widely used pesticides in the world (Balali-Mood and Saber, 2012 ▶). OPs are easily available to the public, particularly in developing countries and thus acute OPs poisoning, either as suicidal attempts or accidentally, is very common (Abdollahi et al., 1997 ▶). World health organization (WHO) reported around 3 million pesticide poisoning, mainly due to OPs each year that resulted in more than 220,000 deaths (Bertolote et al., 2006 ▶).

Atropine and oximes are pharmacological antidotes of OPs, and their efficacy on acute OP poisoning is mostly related to the type of OP, severity of intoxication and the time between OP exposure and antidote administration (Moshiri et al., 2015 ▶). In spite of the standard treatment, around 10% of the patients are expired (Balali-Mood and Saber, 2012 ▶). Therefore, several compounds such as sodium bicarbonate, magnesium sulfate, antioxidants or intravenous lipid emulsions have been evaluated as adjuvant therapy (Amitai et al., 2006 ▶; El-shenawya et al., 2010 ▶; Moshiri et al., 2015 ▶; Moshiri et al., 2013 ▶).

HESA-A is a herbal-marine drug produced in Iran with anti-inflammatory and antioxidants properties and it has been used as adjuvant therapy in some diseases (Ahmadi et al., 2009 ▶; Ahmadi et al., 2005b ▶; Alizadeh et al., 2009 ▶; Barikbin et al., 2010 ▶; Mehrbod et al., 2014 ▶). It is composed of both plant and marine materials, including *Carum carvi* (Persian cumin), *Penaeus latisculatus *(king prawn), and *Apium graveolens *(celery). HESA-A consists of both mineral and organic compounds that contains aminoenthraquinone constituents as well as small amount of water (45, 50 and 5%, respectively) (Moallem et al., 2010 ▶). The exact biological targets of HESA-A have not been determined to date, however, antineoplastic properties of HESA-A in a few end-stage cancer patients, were reported (Ahmadi et al., 2009 ▶; Ahmadi et al., 2010 ▶). It also contains rare elements such as selenium, strontium, zinc, chromium and molybdenum. Low cytotoxicity of HESA-A may be due to the antioxidant properties of some components such as selenium and also due to its components with low toxicity (Fattahi et al., 2017 ▶). 

Roudkenar et al. (2012) ▶ showed that HESA-A had cyto-protective effects *in vitro* and based on the results of antioxidants analysis, it could eliminate free radicals (Roudkenar et al., 2012 ▶). Moreover, according to previous studies on the effects of this natural medication, it was claimed that it can selectively prevent the growth of cancer cells under both clinical and laboratory conditions which is dependent upon its concentration (Schumacher et al., 2011 ▶). In this study, we aimed to evaluate the effect of HESA-A on moderate to severe OPs poisoning. 

## Introduction

Between February 2014 and May 2016, 69 moderate to severe OP poisoned patients who referred to Medical Toxicology Center of Imam Reza hospital, Mashhad, Iran; and had inclusion criteria, were selected. Severity of OP poisoning was estimated based on the following criteria: For moderate poisoning to observe more than 6 of the followings: miosis, loss of consciousness, nausea and vomiting, hypersecretion of saliva, discharge of nose and tracheobronchial tree, abdominal cramp, muscular fasciculation, dysarthria, ataxia, an acetylcholinesterase level equal to 10-20% of the normal level. For severe poisoning to observe more than 5 of the followings: deep coma, pinpoint pupils (which are nonreactive to direct light stimuli), diarrhea, respiratory compromise, pulmonary edema, flaccid paralysis, muscular fasciculation, seizure, heart block, hyperglycemia, and acetylcholinesterase level less than 10% of the normal level. 

The selected patients were randomly divided into two groups of HESA-A treated and control. The control group received the standard treatment only. Due to mortality risk of OP poisoning and ethical points of view, this study was designed as a single blind randomized clinical trial and registered in IRCT (Iranian Registration of Clinical Trial) with registration number: IRCT138811062083N2. All parts of this study were approved by the research ethics committee of Mashhad University of Medical Sciences (MUMS). Since the patients were unaware, his/her legal guardian, read and singed the consent form.

**Figure 1:**
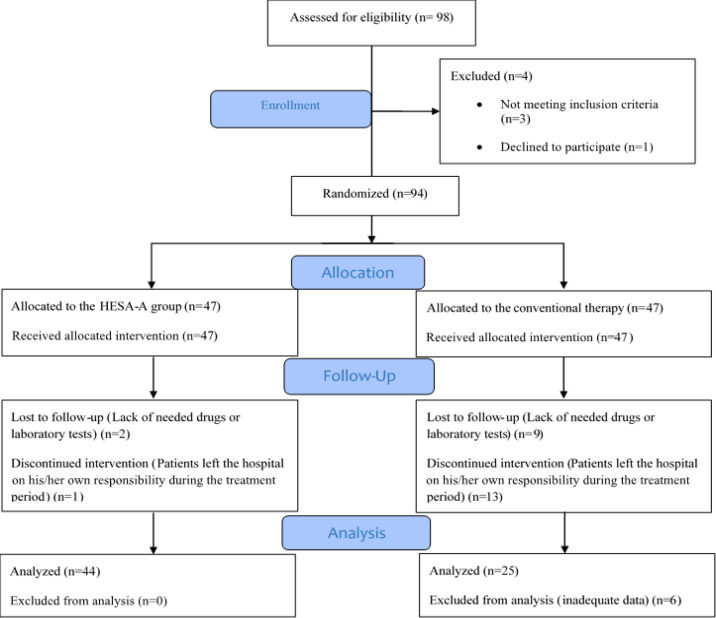
Consort diagram

Exclusion criteria included: 1) Previous history of cardiovascular, neurologic, respiratory, hepatic or renal dysfunctions; 2) Diabetes mellitus; 3) Taking digitalis; 4) Taking CNS depressant medications; 5) Taking neuromuscular receptor blockers; 6) Evidence of co-ingestion of other poisons and/or medications. CONSORT flow diagram of the patients is shown in Figure 1.

All patients were treated by atropine and pralidoxime based on standard protocols of OP poisoning (Bertolote et al., 2006 ▶). Patients were randomly divided into two groups. Patients of the experimental group were treated by HESA-A 50 mg/kg via nasogastric tube, in addition to the standard treatment, daily as long as they had symptoms of organophosphate poisoning. As soon as the patients were symptom-free, HESA-A and atropine administrations were discontinued and the patients were discharged soon afterward. The dosage was selected according to previous studies of Ahmadi and colleagues which reported positive effects of HESA-A on metastatic colon cancer and breast cancer (Ahmadi et al., 2009 ▶; Ahmadi et al., 2005b ▶). HESA-A in the form of powder was provided as a biological product of herbal and marine origin by Dr. Ahmadi to the Medical Toxicology Research Centre (MTRC) of Mashhad University of Medical Sciences (Sadeghi Aliabadi and Ahmadi, 2001 ▶). 

 Serum cholinesterase (S-ChE) and red blood cell cholinesterase (RBC-ChE) of all patients were evaluated before treatment, every morning during the first three days and then every other day until discharge or death.

**Statistical analyses**

All collected clinical and laboratory data were analyzed by SPSS (Version 11.5). Quantitative variables are reported as mean±SD. Qualitative variables are reported as frequency and percentages. The kolmogorov-Smirnov test was used for evaluation of normality of the scores. Student’s t-test was used to compare the means between groups. Chi-square test was used to compare proportional difference in patients' clustering. P-values of less than 0.05 were considered “statistically significant”.

## Results

Out of 69 patients, 44 patients were treated by HESA-A and 25 patients were treated by the standard method Demographic information and frequency of clinical manifestations of the patients are summarized in Table 1. The patients of the two groups were similar in terms of age, gender, percent of urbanization and drug addictions. There were no significant differences in frequencies of clinical manifestations between the two groups (Table 1).

**Table 1.** Demographic information and frequency of clinical manifestations of organophosphorus poisoned patients referred to Medical Toxicology Center of Imam Reza hospital, Mashhad, Iran

**Variable **	**HESA-A treated (44)**	**Control (25)**	**p value**
Age (year) (mean±SD)	28.68±8.8	24.93±9.9	0.12
Gender			
Male Female	27 (61.4%) 17 (38.6%)	11 (40.7.0%) 16 (59.3%)	0.88
urbanization			
City Village	26 (59.1%) 18 (40.9%)	14 (53.8%) 12 (46.2%)	0.38
Level of conciseness (AVPU)*			
Alert Verbal Painful & Coma	24 (54.5%) 12 (27.3%) 8 (18.2%)	19 (70.4%) 5 (18.5%) 3 (11%)	0.42
Addiction	8 (18.2%)	2 (8.7%)	0.22
Diarrhea	14 (31.8%)	11 (40.7%)	0.26
Diaphoresis	35 (79.5%)	19 (70.4%)	0.29
Miosis	32 (72.7%)	14 (51.9%)	0.09
Bradycardia	30 (68.2%)	14 (51.9%)	0.12
Bronchorrhea	21 (47.7%)	13 (48.1%)	0.55
Emesis	31 (70.5%)	13 (48.1%)	0.08
Salivation	37 (84.1%)	18 (69.2%)	0.19
Seizure	4 (9.1%)	1 (3.7%)	0.50
Hypotension	17 (38.6%)	8 (30.8%)	0.39
Fasciculation	13 (29.5%)	6 (22.2%)	0.41
Weakness	33 (75.0%)	22 (81.5%)	0.52

*AVPU: Alert, verbal, pain, unresponsive

Biochemical and toxicological laboratory tests of OP poisoned patients are summarized in Table 2. Among biochemical factors, serum urea was the only one which had a significant correlation with HESA-A administration (p=0.027). Total atropine and pralidoxime administered doses did not show any significant difference between the groups as illustrated in Table 3. Other administered drugs, intensive care unit (ICU) admission rate and duration of hospitalization were similar between the two groups (Table 3).

**Table 2:** Biochemical and toxicological laboratory test results of OP poisoned patients referred to Medical Toxicology Center of Imam Reza hospital, Mashhad, Iran

**Parameter (unite) **	**HESA-A treated**	**Control**	**p value**
Blood Sugar (mg/dl)	137.68±7.27	126.60±8.3	0.28
Urea (mg/dl)	24.0±1.2	19.2±1.7	0.027
Creatinine (mg/dl)	0.93±0.02	0.87±0.04	0.14
Creatinine Phosphokinase (IU/L)	940.43±302.03	369.84±97.54	0.063
Sodium (mg/dl)	139.86±0.6	140.10±0.86	0.83
Potassium (mg/dl)	3.8±0.089	3.6±0.130	0.99
Mean activity of Serum cholinesterase (IU/ml)	6.9±0.83	5.18±0.19	0.11
Mean activity of RBC cholinesterase (IU/ml)	1.08±0.11	0.95±0.08	0.37

**Table 3:** Total administrated doses of atropine and pralidoxime as well as frequency of other drugs, intensive care unit (ICU) admission rate and duration of hospitalization among organophosphate poisoned patients who were treated by HESA-A vs. control group

**Variable**	** unit**	**HESA-A treated**	**Control**	**P value**
Atropine (mean±SD)	mg	110.77±32.95	68.72±8.6	0.08
Pralidoxime (mean±SD)	mg	872.73±327.10	1246±466.9	0.55
Hyoscine	Frequency (%)	12 (27.3%)	6 (30.0%)	0.82
Diazepam	Frequency (%)	7 (15.9%)	5 (25.0%)	0.39
Intubation	Frequency (%)	9 (20.5%)	4 (20.0%)	0.97
ICU admission	Frequency (%)	11 (25.0%)	4 (20.0%)	0.66
Duration of ICU admission (mean±SD)	Day	2.46±0.98	1.20±0.5	0.35
Duration of hospitalization (mean±SD)	Day	7.30±9.0	5.10±0.99	0.25

## Discussion

OPs poisoning is the most common cause of pesticide-induced toxicity in Iran (Abdollahi et al., 1997 ▶; Afshari et al., 2004 ▶). Because of high frequency and morbidity/mortality of OP poisoning, several attempts were made to find an effective adjuvant treatment for the poisoning. For instance, two clinical trials reported positive effects of high doses of bicarbonate and magnesium sulfate, in Iranian and Bangladeshis OP-poisoned patients, respectively (Balali-Mood et al., 2005 ▶; Basher et al., 2013 ▶).

HESA-A is a natural marine compound that is extracted from *Penaeus latisulcatus*, *Carum carvi* and *Apium graveolens* (Vahabpour et al., 2012 ▶). It includes organic constituents (45%), mineral constituents (50%) and water (5%) (Mehrbod et al., 2014 ▶). Its mineral constituents are potassium and magnesium as well as sodium phosphate and calcium carbonate. It also contains a few amounts of zinc, copper, selenium and strontium salts (Barikbin et al., 2010 ▶; Mehrbod et al., 2014 ▶; Vahabpour et al., 2012 ▶). HESA-A was approved by health ministry of Iran as capsule form to be used as adjuvant therapy for treatment of various diseases. Its adjuvant effects in some chronic difficult-to-treat diseases were confirmed in some clinical trials (Mehrbod et al., 2014 ▶). HESA-A has revealed anticancer effect in a dose-dependent manner and inhibited cancer cell growth (Abbasi et al., 2015a ▶; Ahmadi et al., 2005b ▶, 2009; Muhammadnejad et al., 2014 ▶; Sadeghi-Aliabadi and Ahmadi, 2003 ▶). It has also shown valuable effects in patients with metastatic colorectal carcinoma (Ahmadi et al., 2009 ▶; Ahmadi et al., 2010 ▶), breast cancer (Ahmadi et al., 2005b ▶) and oral carcinoma (Abbasi et al., 2015b ▶). It was suggested that these effect of HESA-A are related to the anti-inflammatory and antioxidants effects as well as the presence of calcium and other mineral compounds (Sadeghi-Aliabadi and Ahmadi, 2003 ▶).

OP toxicity revealed severe inflammatory reactions that induce neuropathy (Etemad et al., 2015 ▶). Banks and Lein (2012) ▶ reported that OP toxicity increased inflammatory mediators such as TNFa, IL-1b, and IL-6. They also suggested that chronic inflammation due to OP poisoning causes OP-induced cognition problems (Banks and Lein, 2012 ▶). Moreover, role of anti-inflammatory drugs, ibuprofen and diclofenac sodium in treatment of OP poisoning, was evaluated in animal models (Amitai et al., 2006 ▶). Anti-inflammatory effect of HESA-A was also reported in some studies (Barikbin et al., 2010 ▶). Magnesium, selenium and strontium present in HESA-A, reduce inflammatory cytokines and are effective in wound healing (Alizadeh et al., 2009 ▶). HESA-A has shown some advantages in patients with systemic lupus erythematous and rheumatoid arthritis (Ahmadi et al., 2008 ▶). 

 HESA-A is a known antioxidant (Ahmadi et al., 2005a ▶; Roudkenar et al., 2012 ▶). Some important mechanisms of OP-induced toxicity are activation of oxidative stress pathways, attenuation of total antioxidant capacity and raising lipid peroxides (Etemad et al., 2015 ▶; Moshiri et al., 2012 ▶). Vitamin E, a potent antioxidant, has been reported to have some therapeutic effects in OP-poisoned animals (Etemad et al., 2015 ▶; Moshiri et al., 2012 ▶). HESA-A, at 0.1-0.9 mg/ml concentration, has antioxidant and free radical scavenging effects (Sadeghi-Aliabadi and Ahmadi, 2003 ▶). Despite antioxidant and anti-inflammatory properties of HESA-A, this compound could not reduce atropine or pralidoxime doses required for OP-poisoned patients. It also did not reduce ICU admission rate and duration of hospitalization of the patients as the criteria for morbidity.

The positive effect of HESA-A on psoriatic plaques was time-dependent and thus continued for a long time (Barikbin et al., 2010 ▶). Thus, it was suggested that HESA-A may reduce the OP toxicity in a long period of treatment or it may attenuate delayed complications of OP poisoning. However, in our study, HESA-A did not show any useful effects in treatment of acute OP poisoning.

Serum urea was significantly higher in the HESA-A group than the controls (p=0.027). Serum creatinine level was also higher in the HESA-A group, but did not show any significant difference between the two groups. Higher levels of renal function tests may be due to renal clearance of HESA-A. 

HESA-A contains inorganic substances such as calcium carbonate, potassium, magnesium phosphate and sulfate and elements such as zinc, aluminum, potassium, cobalt, chrome, bromine, iron, and strontium at high concentrations, as well as organic substance (e.g. aminoenthraquinone) (Ahmadi et al., 2010 ▶). These compounds may induce renal dysfunction leading to high urea and creatinine levels. However, renal dysfunction has not been reported so far. 

Nausea and vomiting were more frequent in the HESA-A treated group than the control group. However, this was an initial manifestation and we could not detect any vomiting related to HESA-A administration during the treatment. Overall, HESA-A treated group did not show any significant adverse effect during the treatment. Teratogenic effects of HESA-A has been reported by Moallem and colleagues. They reported embryonic malformations in pregnant rats at higher doses of HESA-A, which are 20 to 40 times higher than the usual therapeutic doses (Moallem et al., 2011 ▶). According to previous studies, no significant adverse effect was reported among patients treated with therapeutic doses of HESA-A (Ahmadi et al., 2005b ▶; Alizadeh et al., 2009 ▶).

 In conclusion, HESA-A did not reduce morbidity and mortality of OPs poisoning and did not induce any major side effect in the patients, except the higher urea and creatinine that may be due to nephrotoxicity of high dose of HESA-A as it was not reported to cause adverse effects at lower doses. Due to higher ICU admission and hospitalization as well as disturbed laboratory tests specifically renal function tests among HESA-A patients, the authors do not recommend administration of HESA-A for acute OP poisoning treatment.
